# Organization and function of neuronal circuits controlling movement

**DOI:** 10.15252/emmm.201607226

**Published:** 2017-01-24

**Authors:** Silvia Arber

**Affiliations:** ^1^ BiozentrumDepartment of Cell BiologyUniversity of BaselBaselSwitzerland; ^2^ Friedrich Miescher Institute for Biomedical Research (FMI)BaselSwitzerland

**Keywords:** Neuroscience

## Abstract

The 2017 Louis‐Jeantet Prize for Medicine winner Silvia Arber describes her laboratory's contributions to our understanding of the neuronal circuits underlying the control of movement in mammals.

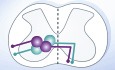

Movement is essential for survival and represents the final behavioral output of many computations in the nervous system. One of the most striking characteristics associated with movement is the seemingly endless repertoire of distinct actions and motor programs that our bodies can generate, raising the important question of the underlying neuronal circuit mechanisms that are at the core of regulating different forms of movement. Motor control‐relevant parameters can be measured throughout the nervous system, indicating that information about movement is broadly distributed. This feature is also underscored by the fact that many diseases affecting the nervous system lead to perturbation in movement, often severely disabling affected patients.

To understand how the various sensory systems functionally assemble and process incoming information, it has been very fruitful in the past to study the organization of neuronal circuits at their first steps into the nervous system. This collective work has unraveled the high precision with which information is processed as well as the identity of involved neuronal subpopulations and their functions. In contrast, much less was known about whether and how motor output pathways at the opposite end of the nervous system follow an organizational logic at the level of neuronal circuits, and how such a circuit logic might translate into different functions in the regulation of movement. This commentary summarizes some of the work my laboratory has contributed recently to the understanding of circuit‐level organizational principles within the final output pathways of the motor system, and how this anatomical work relates to functional parameters in the execution of movement.

## The spinal cord as a highly organized final executive center for body movement

Motor neurons in the spinal cord are spatially organized into motor neuron pools, each innervating a distinct skeletal muscle in the periphery. Since body movement is a result of coordinated muscle contractions, it is essential to understand how different motor neuron pools are recruited in line with the biomechanical ability of the innervated muscles, a property largely determined by the identity of synaptic inputs to these motor neurons. An important question was therefore whether studying the distribution and identity of neuronal populations premotor (i.e., with direct synaptic connections to motor neurons) to functionally distinct groups of motor neurons could be leveraged to visualize and understand the organization of functionally distinct connectivity matrices in the spinal cord. Such approaches were made possible by recent technological advances on genetically modified versions of rabies viruses for transsynaptic tracing, restricting their labeling potential to directly connected (monosynaptic) neuronal populations.

**Figure 1:**
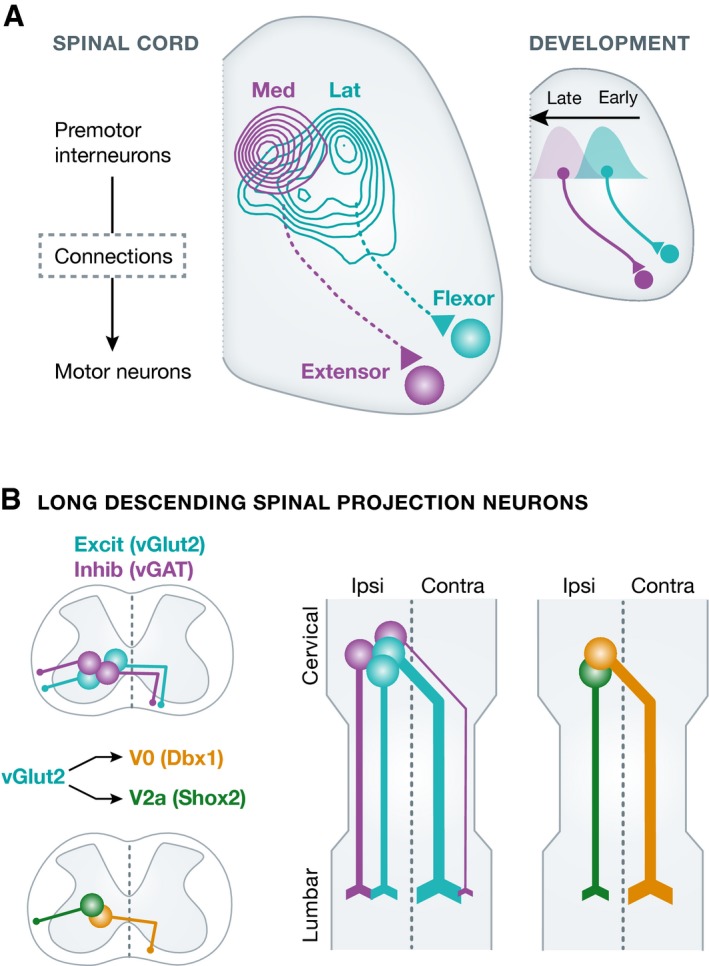
Motor circuit organization in the spinal cord (A) According to functional subdivision by extensor and flexor motor neurons in the spinal cord, connected premotor interneurons segregate along the medio‐lateral axis and by time of neurogenesis during development (adapted from Tripodi *et al*, 2011). (B) Long descending projection neurons in the spinal cord linking cervical and lumbar segments can be subdivided based on neurotransmitter identity (vGlut2, excitatory; vGAT, inhibitory) and developmental origin (V0, Dbx1; V2a, Shox2). These criteria subdivide long projection neurons into different classes also according to projection pattern and synaptic terminations (adapted from Ruder *et al*, 2016).

We applied this emerging technology to reveal the spatial distribution of spinal premotor neurons connected to motor neuron pools of different function (Stepien *et al*, 2010). In this first study, we found that premotor interneurons distribute over many segments of the spinal cord and that patterns are highly reproducible across individuals, but distinct for different motor neuron pools. After this proof‐of‐principle study, we asked whether distinct premotor populations regulate motor neurons responsible for control of stance (extension) and swing (flexion) phases in the locomotor sequence (Tripodi *et al*, 2011). We found that in the overall neuronal distribution pattern, extensor premotor interneurons in the spinal cord are located more medially than their flexor counterparts (Fig 1A), illustrating the existence of an anatomical trace correlating with motor function even at a circuit level only one step away from actual execution. Moreover, the basis for these spatial and connectivity differences is laid down during development, when postmitotic neurons giving rise to extensor and flexor premotor neurons are generated from the same progenitor domain territory but at different developmental time points (Fig 1A; Tripodi *et al*, 2011). In more recent work, we demonstrated that interneurons premotor to motor neurons regulating postural muscles involved in trunk stability, and with very distinct function from limb muscles, show a bilaterally symmetrical distribution in the spinal cord, while counterparts associated with the control of limb muscles are biased toward the ipsilateral spinal cord (Goetz *et al*, 2015). We also found that alternation of axon guidance molecules on genetically defined interneurons downstream of transcription factors can alter premotor connectivity patterns and lead to behavioral abnormalities (Satoh *et al*, 2016). Lastly, the majority of studies on spinal interneurons in the past and in particular the ones linking developmental transcriptional identity to locomotor function focused mostly on local interneuron circuitry (Arber, 2012). In recent work, we describe the diverse genetic identity, synaptic organization, and function of spinal neurons with long axonal projections (Ruder *et al*, 2016). We found that cervical neurons with long descending projections to the lumbar spinal cord subdivide into genetically tractable neuronal subpopulations with distinct connections, in part based on developmental origin (Fig 1B), and that the overall neuronal population plays an important role in the regulation of whole‐body locomotor parameters to ensure stability of locomotion, including regulation of postural stability and speed‐dependent interlimb coordination (Ruder *et al*, 2016). In summary, our work on the organization of motor circuits in the spinal cord reveals that understanding precise connectivity patterns and genetic identities can provide important insight into functional circuit properties in the motor output system within the spinal cord. They raise the question of whether similar principles might apply to descending pathways from the brain, a line of studies we have recently carried out and that is described in the next section.

## The brainstem as modular switchboard for regulation of diverse action programs

While the spinal cord is clearly an essential component for the execution of body movements, it is well established that it cannot generate movement without external input. The most striking demonstration of this point is the observation that patients with complete spinal cord injuries exhibit paralysis of body parts regulated by spinal segments below injury. External input sources to the spinal cord include most importantly descending pathways from the brain and sensory feedback from the periphery. This commentary will solely focus on communication between supraspinal centers in the brainstem and the spinal cord and in particular address the organization of connectivity in relation to behavioral function.

**Figure 2:**
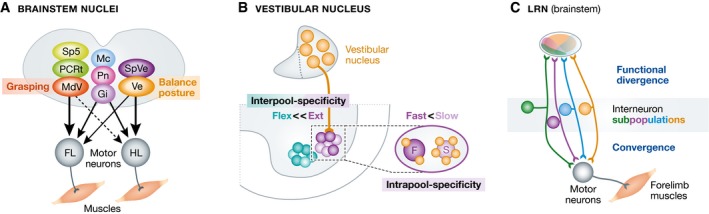
Motor modules in the brainstem (A) Scheme displaying brainstem nuclei with neurons premotor to forelimb (FL) and hindlimb (HL)‐innervating spinal motor neurons in different colors (adapted from Esposito *et al*, 2014); especially highlighted are MdV, needed for efficient forelimb grasping, and the vestibular nucleus (Ve), with a role in posture and balance. (B) Connectivity between neurons in the vestibular nucleus and hindlimb‐innervating motor neurons (adapted from Basaldella *et al*, 2015). Note that Ve input is biased to extensor over flexor motor neurons (interpool specificity) and within the extensor pool to slow over fast motor neurons (intrapool specificity). (C) Subpopulations of functionally distinct spinal interneurons converge on forelimb‐innervating motor neurons, and their ascending projections diverge in the brainstem nucleus lateral reticular nucleus (LRN; adapted from Pivetta *et al*, 2014).

Species with four limbs including humans make extensive use of their extremities, but rostral and caudal extremities show important functional differences, and these are conserved across evolution. The most striking difference is the use of forelimbs for precise manipulation tasks, a functional property almost lacking for hindlimbs in most species. Our experimental approaches to understand whether there are anatomical correlates to these behavioral differences was to uncover sites in the brain in which neurons reside with direct connections to spinal motor neurons innervating forelimb or hindlimb muscles, using transsynaptic rabies virus tracing experiments with monosynaptic restriction (Esposito *et al*, 2014; Fig 2A). Strikingly, we identified more brainstem subregions with neurons establishing direct connections to forelimb than hindlimb‐innervating motor neurons (Fig 2A). In particular, there were three regions that showed almost exclusively connections to fore‐ but not hindlimb‐innervating motor neurons, named MdV, PCRt, and SpV (Esposito *et al*, 2014). Moreover, we identified three regions with indiscriminate connectivity profiles to both kinds of motor neurons and these neurons were found in a bilaterally distributed pattern (Mc, Pn, Gi). And lastly, two brainstem regions showed higher connectivity to hindlimb‐innervating motor neuron populations compared with forelimb counterparts (Ve, SpVe).

To follow up on these anatomy‐based connectivity findings, we started to dissect how subpopulations of brainstem neurons are embedded in motor output pathways and what their functions in the regulation of motor behavior are. We found that within the caudally located MdV brainstem nucleus, only glutamatergic neurons establish synaptic connections to very specific forelimb muscle‐innervating motor neuron pools. In functional studies, we revealed that these excitatory MdV neurons do not have a role in regulating locomotion, but that in the functional absence of these neurons either by specific ablation or by transient pharmacogenetic silencing approaches, mice were significantly impaired in carrying out a forelimb‐reaching and retrieval task for single food pellets. Most notably, we traced the defect specifically to the grasping phase of the unilateral forelimb behavior (Fig 2A), and no deficiency was found in the reaching phase toward the food pellet or the retrieval phase back to the mouth (Esposito *et al*, 2014). A second topic we studied is the connectivity from the vestibular nucleus to hindlimb‐innervating motor neurons (Basaldella *et al*, 2015). As previously found in cats, these synaptic inputs are directed preferentially to extensor over flexor hindlimb‐innervating motor neurons also in mice, but within extensor motor neuron pools, synaptic inputs are targeted in high number to slow‐ over fast type motor neurons (Fig 2B). Since this motor neuron subclass is recruited for postural tasks, the revealed specific connectivity matrix from brainstem vestibular neurons thus matches perfectly this behavioral requirement. We found that the precision of these synaptic connections is established during development and requires multisensory signaling from both vestibular and proprioceptive sources (Basaldella *et al*, 2015). A third example of a functionally dedicated pathway was described in a recent collaborative study, in which we identified specific neurons in the periaqueductal gray in the midbrain involved in the regulation of defensive behavior, signaling through the caudal brainstem nucleus Mc (Tovote *et al*, 2016). Finally, ascending communication from the spinal cord to supraspinal centers is also essential for accurate motor behavior. In this context, we have recently identified a complex connectivity matrix between spinal neurons and the brainstem nucleus LRN, composed of distinct genetically identifiable subpopulations with specific connectivity patterns (Fig 2C; Pivetta *et al*, 2014).

In summary, current evidence from our work and recent studies by other investigators that cannot be described here due to space limitations begin to suggest that the identification of functionally dedicated subpopulations in the brainstem, defined by position, genetic identity, and connectivity, is instrumental to understand the function of these neurons in the regulation of motor behavior. The emerging theme is that these subpopulations and their associated circuitry represent dedicated modules that are at the core of regulating diverse motor actions and programs. Future work will reveal how many of these modules exist, how they interact with each other, and how circuits involved in competing motor programs decide on actual behavior to be carried out.

## Outlook: specific neuronal subpopulations at the core of healthy and diseased nervous system

The theme that anatomical connectivity patterns prefigure behavioral function can be used as an entry point to gain a deeper understanding of the neuronal circuits underlying the regulation of motor behavior, and in particular action diversification. It will be important to understand how motor centers in the brainstem interact with final executive circuits in the spinal cord to implement motor programs for body control, and how higher motor centers involved in decision‐making and action sequence generation interact with neuronal circuits in the brainstem. In the long run, understanding the healthy configuration of these circuits and how they function will likely be very useful for developing strategies to interfere with movement disorders, often affecting higher motor centers, but for which considerable amelioration might be achieved by interference at a level of neuronal circuits closer to execution, including the spinal cord and the brainstem. From our own recent work, we found that a specific source of sensory feedback derived from muscle spindles is absolutely essential to drive functional recovery after incomplete spinal cord injury (Takeoka *et al*, 2014). Notably, the observed functional recovery processes were paralleled by circuit reorganization in the spinal cord establishing detour circuits across the lesion site, and these circuit adjustments were also impaired in the absence of muscle spindle feedback. Together, these findings suggest that not only for the healthy nervous system, but also in disease or after injury, it is crucial to understand the response properties of specific neuronal subpopulations to gain access to key circuit mechanisms to interfere with nervous system dysfunction. I am convinced that future discoveries in this direction will drive the process of developing medicines for the impaired nervous system.

## Conflict of interest

The author declares that she has no conflict of interest.
